# Intensity-modulated pelvic radiation therapy and simultaneous integrated boost to the prostate area in patients with high-risk prostate cancer: a preliminary report of disease control

**DOI:** 10.1002/cam4.278

**Published:** 2014-06-28

**Authors:** Biancamaria Saracino, Maria Grazia Petrongari, Simona Marzi, Vicente Bruzzaniti, Gomellini Sara, Stefano Arcangeli, Giorgio Arcangeli, Paola Pinnarò, Carolina Giordano, Anna Maria Ferraro, Lidia Strigari

**Affiliations:** 1 Department of Radiotherapy, Regina Elena National Cancer InstituteRome, Italy; 2 Medical Physics Laboratory, Regina Elena National Cancer InstituteRome, Italy

**Keywords:** Disease control, IMRT, late toxicity, whole pelvic radiotherapy

## Abstract

The aim of the study was to report the clinical results in patients with high-risk prostate cancer treated with pelvic intensity-modulated radiation therapy (IMRT) and simultaneous integrated boost (SIB) to the prostate area. A total of 110 patients entered our study, 37 patients presented with localized prostate cancer and radiological evidence of node metastases or ≥15% estimated risk of lymph node (LN) involvement, while 73 patients underwent postoperative adjuvant or salvage irradiation for biochemical or residual/recurrent disease, LN metastases, or high risk of harboring nodal metastases. All patients received androgen deprivation therapy (ADT) for 2 years. The median follow-up was 56.5 months. For the whole patient group, the 3- and 5-year freedom from biochemical failure were 82.6% and 74.6%, respectively, with a better outcome in patients treated with radical approach. The 3- and 5-year freedom from local failure were 94.4% and 90.2%, respectively, while the 3- and 5-year distant metastasis-free survival were 87.8% and 81.7%, respectively. For all study patients, the rate of freedom from G2 acute rectal, intestinal, and urinary toxicities was 60%, 77%, and 61%, respectively. There was no G3 acute toxicity, ≥G2 late intestinal toxicity, or G3 late urinary or rectal toxicity. The 3- and 5-year ≥G2 freedom from late rectal toxicity rate were 98% and 95%, respectively, while the 3- and 5-year ≥G2 freedom from late urinary toxicity rate were 95% and 88%, respectively. The study concludes that pelvic IMRT and SIB to the prostatic area in association with 2-year ADT was a well-tolerated technique, providing high disease control in patients with prostate cancer requiring LN treatment.

## Introduction

The real effectiveness of irradiation of the pelvic lymph node (LN) chains in patients treated for high-risk prostate cancer is not yet definitively established, although several studies have been carried out in recent decades. Many problems—such as inadequate total dose to the pelvic field and concomitant androgen deprivation therapy (ADT)—could underlie this lack of clear answers, and may mislead the response evaluation. A major reason is probably that an adequate coverage of the pelvic nodes cannot be obtained using conventional radiotherapy techniques, without a high probability of either acute or late toxicities 1,2.

Three-dimensional (3D) conformal techniques and especially intensity-modulated radiotherapy (IMRT) have shown to be able to modify this scenario, as they allow satisfying coverage of the target, better dose distributions with lower doses to normal tissues and higher doses to the target 3–7. Moreover, IMRT allows the administration of different total doses and doses/fraction to different sides within the irradiation field, according to the “simultaneous integrated boost” (SIB) technique. Therefore, the SIB technique makes it possible to simultaneously give different doses to the prostatic area and the pelvic LN.

The rationale of the pelvic irradiation—either in patients with high risk of subclinical LN involvement (Roach equation ≥15%) 8 or radiologically or pathologically positive nodes—is the probability to improve locoregional control and, possibly, survival, as untreated microscopic or gross residual disease could be a critical source of distant metastases.

In a previous retrospective study 9, we reported mild acute and late enteric, bladder and rectal toxicities, despite an excellent coverage of the target, in patients with prostate cancer and node metastases or estimated risk of node involvement ≥15%, using pelvic IMRT with SIB to the prostatic area.

The aim of the present study was to evaluate biochemical and clinical control and late toxicity in a larger cohort of patients who had matured a sufficient follow-up and completed prescribed ADT.

## Materials and Methods

Eligibility criteria included both patients with localized prostate cancer and radiological evidence of LN metastases or estimated risk of LN involvement ≥15%, according to Roach equation 8, and patients undergoing radical prostatectomy (RP) with adverse risk factors, biochemical or macroscopic residual or recurrent disease, and histologically proven LN metastases or risk of nodal involvement >15%. No patients had detectable distant metastases.

The adverse risk factors taken into account for patients undergoing RP were initial prostate-specific antigen (iPSA), pathological stage and Gleason score, extracapsular extension, surgical margins status, and postoperative and pre-RT PSA level. The pretreatment clinical and pathological features of the three groups of patients are detailed in Table 1.

**Table 1:** Patient and tumor characteristics

	All patients	RAD	ART	SRT	*P*-value
Patients (*n*)	110	37	38	35	
Age (years)					
Median	67	68	63	67	
Range	47–83	50–83	47–77	57–76	0.004
T-stage					
T1–T2	38	19	7	12	
T3–T4	72	18	31	23	0.007
i-PSA					
<10	46	10	16	20	
10–20	33	10	12	11	0.028
>20	31	17	10	4	
GS					
2–6	11	5	2	4	
7	55	14	24	17	0.335
8–10	44	18	12	14	
N					
N0	64	27	14	23	
N1	26	10	10	6	<0.001
Nx	20	0	14	6	
Risk					
0 < *r* < 15	22	5	7	10	
15 ≤ *r* <30	54	15	19	20	0.064
30 ≤ *r*	34	17	12	5	

RAD, radical radiotherapy; ART, adjuvant radiotherapy; SRT, salvage radiotherapy; i-PSA, initial prostate-specific antigen; GS, Gleason score.

Thirty-seven patients with histologically confirmed prostate adenocarcinoma were treated with radical radiotherapy (RAD) to the prostate and LN, and ADT consisting of either an luteinizing hormone-releasing (LH-RH) analog and nonsteroidal antiandrogens or the LH-RH analog alone. ADT was started 2 months before irradiation and continued for 2 years. Seventy-three patients received postoperative adjuvant (ART) or salvage (SRT) radiotherapy in combination with the same hormone treatment.

### Pretreatment evaluation

Pretreatment evaluation included medical history, physical and digital rectal examination, hematologic and PSA assays, transrectal ultrasound and/or magnetic resonance imaging, abdomino-pelvic computed tomography, and bone scan. Sextant prostate biopsies were performed in all patients undergoing RAD.

### Radiotherapy

Patients were simulated in prone position with a customized cradle. An axial CT-scan with 5-mm-thick slices was obtained from 10 cm above the common iliac artery bifurcation to 5 cm below the ischial tuberosities, after administration of intravenous contrast medium to allow a better delineation of the LN areas. Patients were instructed to void their bladder no less than 1.5 h before the simulation, and before each treatment.

An enema before simulation and a light laxative and dietary measures during the radiotherapy course were recommended only for patients with habitual constipation.

In patients undergoing RAD, the clinical target volumes CTV1 (prostate and seminal vesicles) and CTV2 (common, internal and external iliac, presacral, and obturator nodes), the planning target volumes PTV1 (generated by a 0.6-cm expansion posteriorly and a 1-cm expansion in all other directions, around the CTV1), PTV2 (by adding 1 cm around the CTV2), and the OARs (rectum, bladder, femoral heads, and intestinal loops) were outlined on each CT slice. For the patients who had previously undergone RP, the CTV1 included the prostate bed.

A commercial inverse planning software (Eclipse v.8.9 Varian Medical Systems, Palo Alto, CA) was used to generate IMRT plans, using a five-field SIB technique.

The prescribed dose to the PTV1 ranged from 76 to 80 Gy (median: 80 Gy) in 38/40 fractions for RAD treatment, from 66 to 74 Gy (median: 70 Gy) in 33/37 fractions for ART, and from 64 to 76 Gy (median: 73 Gy) in 32/38 fractions for SRT group. The prescribed dose to the PTV2 ranged from 53 to 61 Gy (median: 57 Gy) for RAD group, from 49 to 58 Gy (median: 54 Gy) for ART group, and from 52 to 58 Gy (median 55 Gy) for SRT group (Table 2).

**Table 2:** Dose/volume data

	All patients	RAD	ART	SRT
PTV1 volume (cc), median (range)	171 (102–275)	176 (115–238)	168 (102–240)	169 (109–275)
PTV2 volume (cc), median (range)	806 (648–1228)	769 (649–1097)	782 (657–1065)	873 (648–1228)
PTV1 dose (Gy), median (range)	74 (64–80)	80 (75–80)	70 (66–76)	73 (64–78)
PTV2 dose (Gy), median (range)	55 (49–61)	57 (53–61)	54 (49–58)	55 (52–58)

RAD, radical radiotherapy; ART, adjuvant radiotherapy; SRT, salvage radiotherapy.

The total dose to the prostate area was delivered in 2 Gy/fraction, while the pelvic LN chains received lower doses per fraction, with a median value of 1.56 Gy (95% confidence interval of the median: [1.54, 1.61 Gy]). The total dose was adjusted to be equivalent to about 50–52 Gy given in 2 Gy fractions (NTD2), assuming an *α*/*β* ratio of 1.5 10.

The dose was calculated so that the 90% of PTVs (D90) received the prescribed dose. The dose inside the PTV1 ranged from 90% to 107%, while the PTV2 received a more inhomogeneous dose distribution, given the close proximity to the PTV1.

The constraints for the OARs were defined as follows, based on our previous clinical experience 9,11 and before Quantec Publication: no more than 60%, 50%, and 30% of the rectal wall received >40 Gy (V40), 50 Gy (V50), and 70 Gy(V70), respectively. After Quantec Publication, the constraints for rectum were no more than 50%, 35%, 25%, 20%, and 15% of the rectum received >50 Gy (V50), 60 Gy (V60), 65 Gy (V65), 70 Gy (V70), and 75 Gy (V75), respectively, according to the Quantec recommendations 12. No more than 70% and 50% of the bladder received >50 Gy (V50) and 70 Gy (V70), respectively; no more than 5% and 50% of the intestinal loops received >55 Gy (V55) and 40 Gy (V40), respectively. Up to 5% of the femoral heads received 50 Gy (V50).

Treatment was performed by 15 MV photon beams using Varian 2100 linear accelerators equipped with 120-leaf collimators (Varian Associates, Palo Alto, CA), in sliding window modality. The reproducibility of the treatment was daily verified by using orthogonal portal images. All patients provided written informed consent.

The primary endpoint of our study was the evaluation of the biochemical and relapse-free survival in patients who had completed their treatment. Secondary endpoints were acute and late toxicities (scored according to the RTOG scale v3), overall survival (OS), local failure, and metastases-free survival. Biochemical failure was defined according to the RTOG-ASTRO Phoenix Criteria (nadir-PSA + 2 ng/mL) 13.

### Statistical analysis

Tests for statistical significance were performed with the chi-square and *t*-test for categorical and continuous variables, respectively.

All times were calculated from the first day of radiotherapy. For all measured endpoints, patients were censored at the time of the specific event. The actuarial curves of the length of time until late toxicity, biochemical failure, and local or distant recurrence were calculated by the Kaplan–Meier method. The differences between the actuarial curves were evaluated by the log-rank test. To compare groups, the median values of dosimetric/volumetric data were taken as cutoff, while in the multivariate analysis they were considered as continuous variables.

A multivariate Cox proportional hazards model was carried out to assess the relative influence of prognostic factors on survival. Enter and remove limits were *P* = 0.10 and 0.15, respectively. The assessment of interactions between significant investigation variables was taken into account when developing the multivariate model. All of the tests were two-sided and *P* < 0.05 was considered significant.

## Results

Between November 2003 and November 2010, 110 patients with a median age of 67 years (range: 47–83) were treated with concomitant irradiation of the prostate or prostate bed and pelvic nodes, by means of IMRT and SIB technique. Thirty-seven patients were treated with RAD approach, 38 underwent postoperative ART, and 35 SRT treatment. The median follow-up was 56.5 months (range 20–97).

### Acute toxicity

The rate of patients free from G2 acute rectal, intestinal, and urinary toxicities was 60%, 77%, and 61% of patients, respectively, and none experienced G3 toxicities (Table 3). The G2 acute urinary toxicities were significantly lower in ART or SRT treatment than in RAD group, because of smaller volumes of bladder receiving high doses (toxicity-free rates of 70%, 82% vs. 32%, respectively; *P* < 0.001) (Table 3). No significant difference was observed for G2 intestinal or rectal toxicity among the three treatment groups.

**Table 3:** Freedom from acute toxicity

Freedom from acute toxicity	All patients (%)	RAD (%)	ART (%)	SRT (%)	*P*-value
Rectal	60	62	58	61	0.913
Intestinal	77	81	70	82	0.387
Urinary	61	32	70	82	0.001

RAD, radical radiotherapy; ART, adjuvant radiotherapy; SRT, salvage radiotherapy.

Acute urinary, intestinal, and rectal toxicities did not significantly correlate with either the prostate or pelvic dose administered.

The toxicity analysis confirmed that tolerability was as good as shown in our previous toxicity study 9.

### Late toxicity

The analysis of late toxicity showed a rate of freedom from G2 late rectal or urinary toxicity at 5 years of 94.6% and 88.4%, respectively. No ≥G2 late intestinal toxicity was observed.

**Figure 1:**
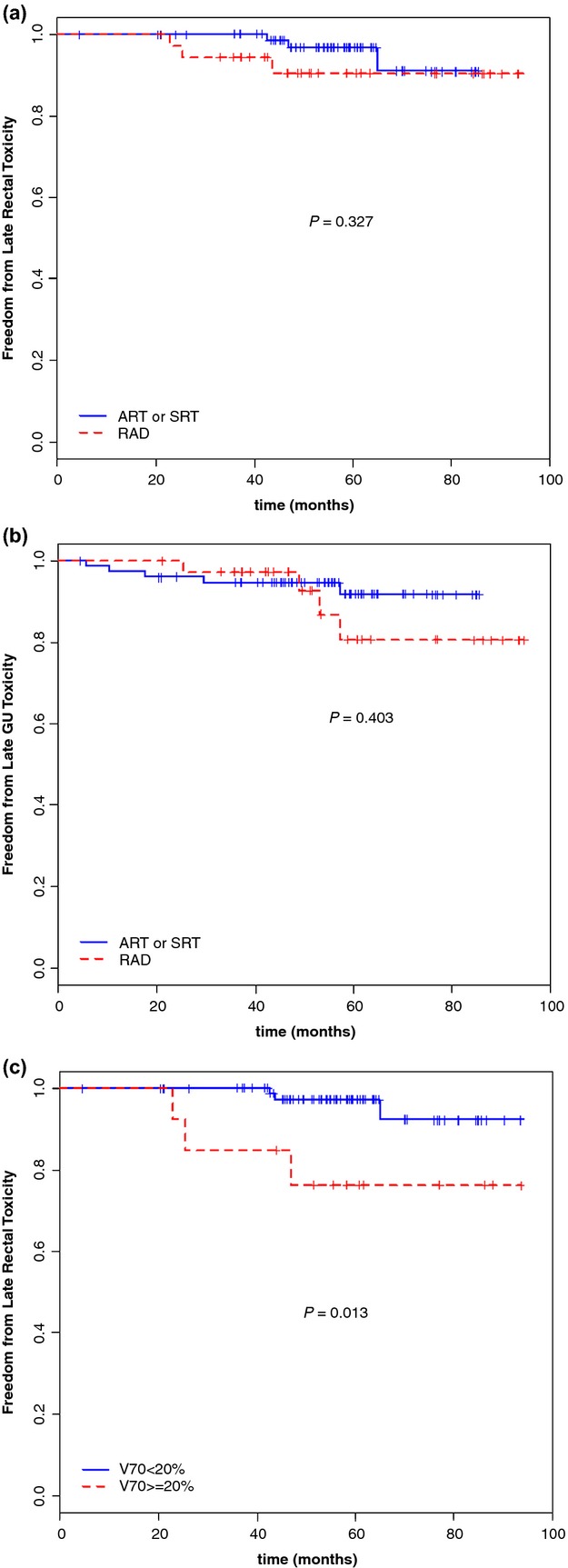
Actuarial analysis of the freedom from grade 2 late rectal toxicity for radical (RAD) versus postoperative irradiation (ART or SRT intent) (A). Actuarial analysis of the freedom from grade 2 late genitourinary (GU) toxicity for radical (RAD) versus postoperative irradiation (ART or SRT intent) (B). Actuarial analysis of the freedom from grade 2 late rectal toxicity for radical (RAD) versus postoperative irradiation (ART or SRT intent) in relation to the rectal volume receiving doses >70 Gy (C).

None of the patients experienced late G3 urinary or rectal toxicity. At 5 years, the actuarial rates of freedom from G2 rectal late toxicity were 90.4% and 96.6% for the RAD and ART/SRT groups, respectively (Fig.1A), while the 5-year freedom from G2 late urinary toxicity were 80.5% and 91.6%, respectively (Fig.1B). Late toxicity was not significantly affected by prostatic or pelvic irradiated volume, while a significant lower G2 rectal late toxicity was observed in patients when less than 20% rectum volume received doses higher than 70 Gy (Fig.1C, *P* = 0.013).

### Overall and biochemical progression-free survival

**Figure 2:**
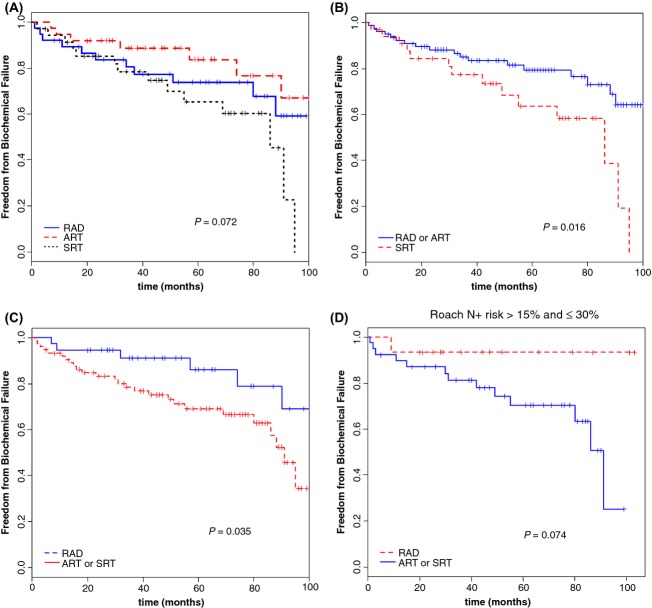
Freedom from biochemical failure for the three patient groups, according to the treatment type (A–C) and in the subgroup with intermediate risk (>15% and ≤30%) (D).

For the whole patient group, the 3- and 5-year freedom from biochemical failure (FFBF) were 82.6% and 74.6%, respectively. A trend to significance were found among the radical, adjuvant, or salvage approaches, with 5-year actuarial rates of 83.7%, 73.8%, and 65.3%, respectively (*P* = 0.072) (Table 4, Fig.2A).

**Table 4:** Freedom from biochemical failure, local failure, distant metastases, and overall survival at 3 and 5 years

Group	FFBF		FFLF		DMFS		OS	
			
	3 years	5 years	3 years	5 years	3 years	5 years	3 years	5 years
All patients	82.6	74.6	94.4	90.2	87.8	81.7	96.3	88.1
RAD	88.6	83.7	97.3	97.3	91.9	87.1	97.3	87.3
ART	80.5	73.8	97.1	90.0	91.6	88.1	94.7	88.6
SRT	78.4	65.3	88.5	83.6	79.4	68.8	97.1	87.6

RAD, radical radiotherapy; ART, adjuvant radiotherapy; SRT, salvage radiotherapy; FFBF, freedom from biochemical failure; FFLF, freedom from local failure; DMFS, distant metastasis-free survival; OS, overall survival.

Actuarial analysis confirmed the benefit of the RAD and ART versus the salvage treatment (*P* = 0.016, Fig.2B), as well as the radical approach compared to the others (*P* = 0.035, Fig.2C).

The analysis of FFBF according to the risk of node involvement did not show any statistically significant differences, but in the subgroup with an ‘intermediate risk” (>15% and ≤30%), a trend to significance was observed for the RAD group versus the ART and SRT treatments (*P* = 0.074) (Fig.2D).

Considering the whole group, the 3- and 5-year freedom from local failure (FFLF) were 94.4% and 90.2%, respectively, without any differences among the three treatment approaches (*P* = 0.224) (Table 4).

Of the 26 patients with N1, seven developed distant metastases (two of whom with concomitant extra-regional LN recurrence, outside of radiation field) and three showed biochemical relapse, while 16 (61.5%) patients achieved a complete response and are still alive without biochemical and/or detectable disease.

**Figure 3:**
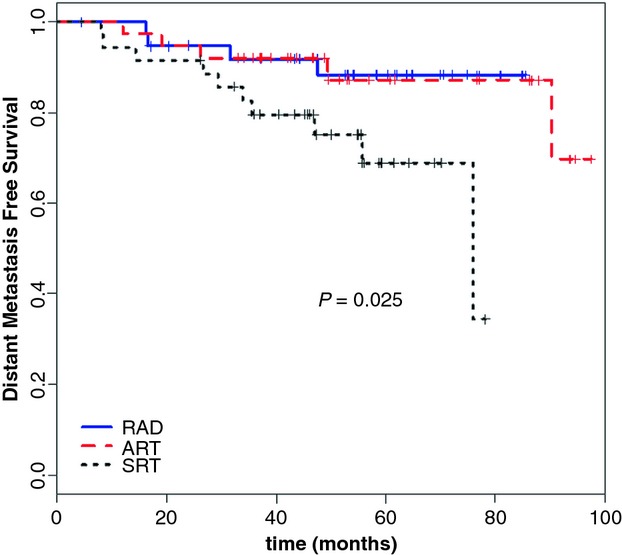
Distant metastasis-free survival according to the treatment type.

In the whole group, the bone was the most frequent localization of metastatic disease (10 patients). The rate of freedom from distant metastases was similar for RAD and ADT, while was significantly lower for SRT (*P* = 0.025) (Fig.3).

Multivariate analysis confirmed the salvage intent (*β* = 1.4910, SE = 0.8494, *P* = 0.08) and LN risk involvement (*β* = 0.0210, SE = 0.0093, *P* = 0.01) as prognostic factors for biochemical relapse. No other variables (PTV1/PTV2 dose or volume) were significantly related to FFBF.

**Figure 4:**
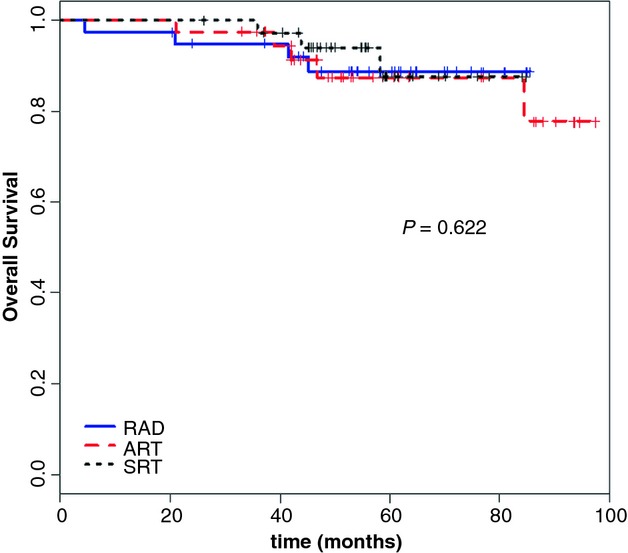
Overall survival according to the treatment type.

For the whole patient population, the 3- and 5-year OS were 96.3% (97.3% for RAD, 94.7% for ART, and 97.1% for SRT) and 88.1% (87.3%, 88.6%, and 87.6%, for the three groups), respectively (*P* = 0.622) (Fig.4, Table 4). The 3- and 5-year DMFS were 87.8% (91.9% for RAD, 91.6% for ART, and 79.4% for SRT) and 81.7% (87.1%, 88.1%, and 68.8%), respectively (Table 4).

## Discussion

In patients with high-risk prostate cancer, there is a high probability of subclinical LN risk involvement that can be estimated using the Roach equation 8. Although the real role of pelvic irradiation is still controversial, in the current clinical practice the treatment of pelvic nodes is generally employed when the predicted risk of involvement is ≥15%.

The rationale for the elective pelvic node treatment is that it may improve the disease-free survival in a subset of patients with occult node involvement, in absence of distant metastases. Probably, the evidence of the benefit of pelvic irradiation has not yet been clearly shown, because the conventional radiation techniques do not allow adequate pelvic nodes coverage through the delivery of sufficiently high doses, as these could exceed the tolerance of normal tissues, with consequent unacceptable side effects 1,2.

Furthermore, patients with high-risk prostate cancer are generally treated with long-term ADT that might hide the real impact of radiotherapy on disease control. In an update analysis of RTOG 94-13 phase III trial, Lawton et al. showed an unexpected and interesting interaction between ADT and radiation, probably due to the immunomodulation induced by the ADT, according to Mercader et al. who reported a T-cell infiltration induced by antiandrogen drugs and a consequent increase in apoptosis. The T-cell infiltration within the involved nodes could improve the efficacy of the RT 14–16.

The 11c-choline PET, spectroscopy and diffusion-weighted MRI are useful for detecting and locating primary or recurrent disease and involved LN, both in staging and in posttreatment evaluation 17–19. The latest diagnostic procedures, such as lymphotropic nanoparticle-enhanced MRI and MRI lymphangiography could reduce the pretreatment underestimation of the LN involvement and, hence, the stage migration that so strongly bias the comparison between radiotherapy and surgery results 20–22.

IMRT is increasingly becoming the most used radiation technique for prostate cancer, providing optimized dose distributions with decreased volumes of OARs receiving high doses and higher doses to both the prostatic area and the pelvic LN. Moreover, IMRT allows the safe delivery of SIB to any microscopically or macroscopically involved area.

The use of safely administered higher doses has opened a new therapeutic scenario for all malignancies in which locoregional control plays a key role. As the probability of locoregional failure and disease spreading is closely related to risk factors, the remarkable pathological upgrade after RP versus the preoperative findings, and the consequent translation into a worse risk category, corroborate the probability of subclinical involvement in patients with adverse risk factors, and also explain the not infrequent finding of unexpected pathological node metastases.

Several studies based on surgical series showed that a standard LN dissection (SLD) could greatly underestimate the LN involvement 23–25. The lymphoscintigraphy and sentinel lymph-nodes identification has strengthened the prognostic and therapeutic value of the extended pelvic LN dissection (ELD), stressing the importance of large irradiation fields and techniques that allow to safely administer high doses, and support the use of pelvic irradiation after a RP with SLD, both with adjuvant and salvage intent.

In our study, we used an intentional inhomogeneous dose distribution employing IMRT, simultaneously treating both the prostate area with conventional dose/fraction and the pelvic nodes with total doses equivalent to 50–52 Gy (assuming an *α*/*β* ratio of 1.5 Gy) and a lower dose/fraction. This technique not only generates concave dose distributions, with considerable geographic sparing of bowel loops and rectum, but also delivers a low dose/fraction to the OARs, probably representing another mechanism of better tolerance.

As a matter of fact, despite the high doses administered to the prostate area, both in RAD and in postoperative treatments, the toxicity analysis confirmed low acute and late genitourinary, enteric and rectal rates as previously reported 9.

Furthermore, despite the total dose to the pelvis (equivalent to 50–52 Gy when we assume an *α*/*β* ratio of 1.5 Gy), seems a mild dose escalation, the encouraging disease control rates lead to hypothesize that subclinical or detectable LNs metastases could have a different radiobiological behavior from the primary lesion, being more aggressive and showing a major cellular replication and metastatic potential. Based on this supposition, the *α*/*β* ratio to take in consideration for LN metastases is likely to be higher than 1.5 Gy and, consequently, the really administered equivalent dose to the pelvis should be greater, realizing an actual dose escalation.

Of the 26 patients with positive LNs, after a median follow-up of 60 months, 16 (61.5%) patients showed a complete response.

For the whole patient group, the 3- and 5-year FFBF were 82.6% and 74.6%, respectively. A statistically significant different FFBF was observed in patients undergoing radical, adjuvant, or salvage radiotherapy with 5-year actuarial rates of 83.7%, 73.8%, and 65.3%, respectively. A statistically significant advantage was also confirmed between patients undergoing RAD or ART versus SRT group.

A positive trend for FFBF was observed in the subgroup with an “intermediate risk” of node involvement (>15% and ≤30%), in which a statistical benefit was found for RAD group. These results suggest that these patients could benefit more from pelvic irradiation, according to Seaward et al. 26, probably because patients with higher node involvement risk and, therefore, worse prognosis, could already have occult distant metastases.

The worse outcome for patients undergoing SRT treatment, in the whole patient group and in the “intermediate-risk” subgroup, underline the impact of pre- and postoperative risk factors and locoregional control on disease outcome, in accordance with several randomized trials, and confirming that a better selection of patients may lead to a better therapeutic choice 27.

These results may highlight a better outcome for patients treated with elective radiotherapy and ADT versus a surgical approach, providing clinical benefit in terms of FFBF according to several large studies.

The rates of FFLF were elevated (94.4% and 90.2% at 3 and 5 years, respectively) and were not significantly different among the three therapeutic approaches, proving that the use of surgical procedures did not seem to increase the locoregional control.

The 3- and 5-year OS were 96% and 86%, respectively. Only three patients died from disease progression.

The actuarial analysis of the 3- and 5-year DMFS were 87.8% and 81.7%, without significant difference between RAD and ADT approaches, but significantly lower for SRT group (*P* = 0.025) (Fig.3).

At a median follow up of 56.5 months, in quite a large cohort, our findings are consistent with the best results reported in literature using radiotherapy and ADT in high-risk prostate cancer, with similar or even higher FFBF and OS rates 14,15,27–32.

As local control is one of the stronger predictors of disease outcome, also in terms of DMFS and CSS, the administration of higher radiation doses with an optimal coverage of the areas of clinical or subclinical disease appears to be a crucial point. Nevertheless, further meaningful information should be provided by the on-going phase III RTOG 0534 and phase III RTOG 0924 trials.

Even if the lack of randomization might limit our work, our findings suggest that the technique employed substantially reduces the frequency and severity of acute and late toxicity, as reported in a previous feasibility study 9. Moreover, the high doses administered to the prostatic area and pelvic LN seem to assure a high probability of locoregional control, as demonstrated by the significant percentage of FFBF and OS in locally advanced disease.

## Conclusions

Our results showed very interesting disease control rates when using high-dose irradiation on whole pelvic and SIB to the prostatic area, in association with long-term ADT, confirming the good tolerance of the employed treatment modality. These encouraging findings have led us to introduce this therapeutic approach in our current clinical practice, in order to definitively evaluate its potential benefit in an even larger cohort with a longer follow-up.
